# DDS: integrating data analytics transformations in task-based workflows

**DOI:** 10.12688/openreseurope.14569.2

**Published:** 2023-04-11

**Authors:** Nihad Mammadli, Jorge Ejarque, Javier Alvarez, Rosa M. Badia

**Affiliations:** 1 Workflows and Distributed Computing, Barcelona Supercomputing Center, Barcelona, Catalunya, 08034, Spain; 2 IOVLabs, Gibraltar, Gibraltar

**Keywords:** Big Data High Performance, Data Analytics, Parallel Computing, Task Based Programming Models

## Abstract

High-performance data analytics (HPDA) is a current trend in e-science research that aims to integrate traditional HPC with recent data analytic frameworks. Most of the work done in this field has focused on improving data analytic frameworks by implementing their engines on top of HPC technologies such as Message Passing Interface. However, there is a lack of integration from an application development perspective. HPC workflows have their own parallel programming models, while data analytic (DA) algorithms are mainly implemented using data transformations and executed with frameworks like Spark. Task-based programming models (TBPMs) are a very efficient approach for implementing HPC workflows. Data analytic transformations can also be decomposed as a set of tasks and implemented with a task-based programming model.

In this paper, we present a methodology to develop HPDA applications on top of TBPMs that allow developers to combine HPC workflows and data analytic transformations seamlessly. A prototype of this approach has been implemented on top of the PyCOMPSs task-based programming model to validate two aspects: HPDA applications can be seamlessly developed and have better performance than Spark. We compare our results using different programs. Finally, we conclude with the idea of integrating DA into HPC applications and evaluation of our method against Spark.

## I. Introduction

High-performance computing (HPC) provides computational resources, software environments and programming models to enable the execution of large-scale e-science applications, *i.e.*, with the objective of making predictions or simulations such as weather forecasting or protein interaction modelling. Recently, with the introduction of Big Data technologies, e-science applications have evolved to more complex workflows where traditional HPC simulations are combined with data analytic (DA) algorithms. However, implementing applications that combines both aspects requires a lot of engineering efforts in terms of deployment and of integration of the HPC and data analytic aspects. For HPC workflows, developers use parallel programming models, while DA algorithms are mainly implemented using DA transformations using frameworks such as Spark ^1^. In addition, some glue code that coordinates the execution and exchanges of data between the application components needs to be implemented. At deployment and operation phase, the work doubles since both HPC and Big Data environments have to be installed, configured and run at the same time. Furthermore, Big Data frameworks have been designed to run in traditional data centres, and they do not get benefit from the specific HPC hardware, such as high-speed networks.

High-performance data analytics (HPDA) is a current trend in e-science research which aims at the integration of traditional HPC with the recent DA frameworks ^2^. Most of the work done until now in this field has focused on improving the data analytic frameworks by implementing their engines on top of HPC technologies such as MPI to benefit from the HPC hardware and speed up the execution of DA algorithms ^3, 4^. However, as raised above, there is still a lack of integration from the programming interface point of view. In the literature of HPC programming models we find task-based programming models. These models provide a good abstraction for developers and they are an efficient approach for implementing parallel HPC workflows. On the other side, DA algorithms are mainly programmed by applying *transformations* and *actions* in a dataset (a set of data elements). Transformations are functions which are applied for each element of the dataset (without modifying the number of elements) and actions are functions which are applied to the whole dataset and can modify the number of elements. To efficiently parallelise these algorithms, the dataset is divided into partitions (a group of elements), and transformations and actions are applied to these partitions. Therefore, data analytic algorithms can be decomposed as a set of tasks on top of task-based programming models.

In this paper, we present a methodology to develop HPDA applications on top of a task-based programming model. We propose the Distributed Data Set (DDS), an implementation of the data analytic transformations and actions on top of TBPMs. It allows developers to combine HPC workflows and data analytic transformations in a seamless way, developed as a single application, without requiring the use and deployment of several frameworks and achieving good performance. A prototype of this approach has been implemented on top of the PyCOMPSs task-based programming model. The prototype has been validated from a functional point of view by implementing a complex workflow that combines different DA transformations with other computational tasks. Moreover, we have executed big data benchmarks on top of the prototype to compare its performance with PySpark, which outperforms. The rest of the paper is organized as follows: Section II presents the related work; Section III introduces the proposed methodology to seamless integrate HPC workflows and data analytic algorithms; and Section IV describes how it has been implemented on top of PyCOMPSs. Then, Section V presents the evaluation; Finally, Section VI draws the conclusions.

## II. Related work

HPC applications are implemented using parallel programming models, which allow developers to efficiently execute applications leveraging the supercomputer architecture. They are divided into two groups: shared memory models such as OpenMP ^5^ focus on the intranode parallelism; and distributed memory models that support applications whose parallelism involves multiple computing nodes, such as MPI ^6^ or Partitioned Global Address Space (PGAS). PGAS programming models assume a global memory address space logically partitioned and a portion of it is local to each process (e.g. UPC ^7^ or GASPI ^8^). Another interesting approach for HPC is the task-based programming model which can be applied either in shared or in distributed environments and provides a good trade-off between abstraction and performance. Since version 3.0, OpenMP supports tasking parallelism. Other programming models which allow this model are OmpSs ^9^, StarPU ^10^, Legion ^11^ or COMPSs ^12^ for distributed environments.

Regarding Big Data applications, Spark ^1^ and MapReduce ^13^ are today’s well-known frameworks. Spark provides in-memory DA operations for several programming languages. Its Resilient Distributed Dataset (RDD) ^14^ high-level abstraction has a rich set of methods for DA applications and eases the development for distributed data. On the other hand, MapReduce provides a programming model where developers define the computation with the map and reduce functions, and the parallelization is automatically done by the underlying runtime.

Most of the previous work on integrating Big Data and HPC are based on the implementation of Big data frameworks on top of the HPC technologies, especially on top of MPI. For instance, Alchemist ^3^, Spark-DIY ^4^ and Harp ^15^ allow users to call MPI-based libraries directly from Spark applications allowing users to substitute inefficient computations with calls to efficient MPI-based implementations. The main advantage of these approaches is that they keep Spark as a programming model, so users do not need to change their codes. Other approaches like Dask ^16^ offer a client API which can be used to implement big data applications together with other complex workflows. Some in situ high-performance workflows such as Decaf ^17^ provide a high-level Python API for the definition of workflow graphs, but require an additional implementation when used with other DA tools like Spark. It could be interesting for new applications but it requires a refactor for existing applications. Finally, Twister2 ^18^ focuses on redesigning the whole big-data stack in order to get a balance between performance and usability which allows the inclusion of proven HPC existing technologies in a unified environment. In our approach, we have focused on the integration of HPC and Big Data technologies from a programming point of view, allowing developers to create applications which combine different types of algorithms in a single environment.

## III. Methodology

**Figure 1.**
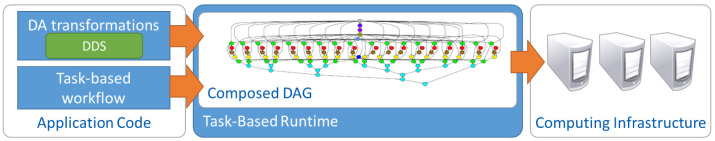
Overview of the proposed integration.

This section presents the methodology proposed for programming HPDA applications on top of a task-based programming model. Figure 1 shows the overall approach of the methodology. On the left, we have the application code, composed of a part performing DA transformations and another part executing parallel code, implemented as task-based workflows. To allow users to seamlessly integrate data transformations in task-based workflows, we propose the Distributed Data Set (DDS). DDS is a library that provides an implementation of data transformations and actions (such as the ones defined in RDD’s Spark’s API) using task-based parallelism. Each transformation and action defined in the API will generate a task-dependency graph composed not only of other transformation graphs (such as in Spark) but also of graphs generated by the rest of the application. As depicted in the figure, the task-based runtime gets tasks from all application parts, generating a composed graph that includes the dependencies between the different parts. The generated Directed Acyclic Graph (DAG) of the whole application will be scheduled and executed in the available computing resources. We expect the following benefits:

- Developers do not need to perform significant changes to the DA algorithms since the API will almost be the same, and integrating them with other computations can be as easy as passing data between task invocations
- Since a DAG is generated for the whole application, there is no need for synchronization between DA parts and the rest of the parallel regions. The runtime will manage the data dependencies and data movements, avoiding unnecessary global synchronizations.
- Thanks to the composed DAG, the runtime will do better resource management, overlapping reduction regions whose resource usage is low with other independent parallel regions where the resource demand is higher.

**Figure 2.**
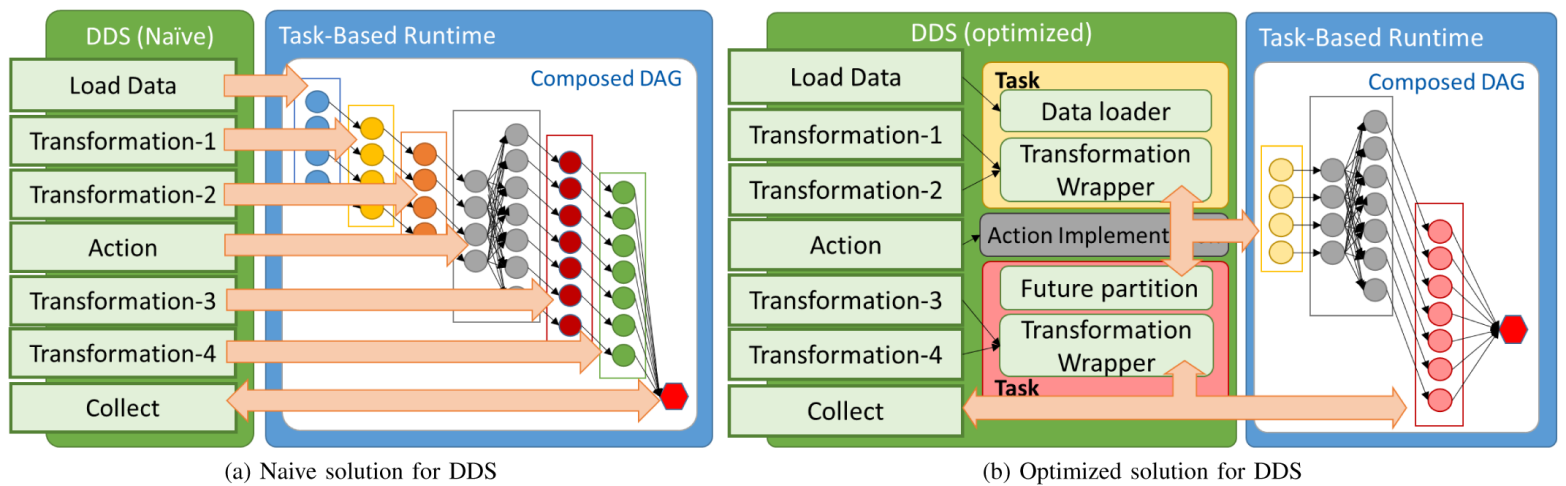
Distributed Data Set approaches.

A naïve approach for DDS can be proposed by creating a separate workflow of tasks for each transformation or action method that the user invokes. In this case, as described in Figure 2a, when the user calls a DDS method a set of tasks (one per partition) is added to the DAG for each transformation and a workflow of tasks per action. This approach has two main disadvantages: first, every new DDS method included in the library requires the implementation of a specific workflow. This requires a lot of effort for implementing and maintaining DDS. Second, DDS will create a new set of tasks for each method, which implies a high overhead to manage many tasks at runtime and to distribute them between the computing nodes.

**Figure 3.**
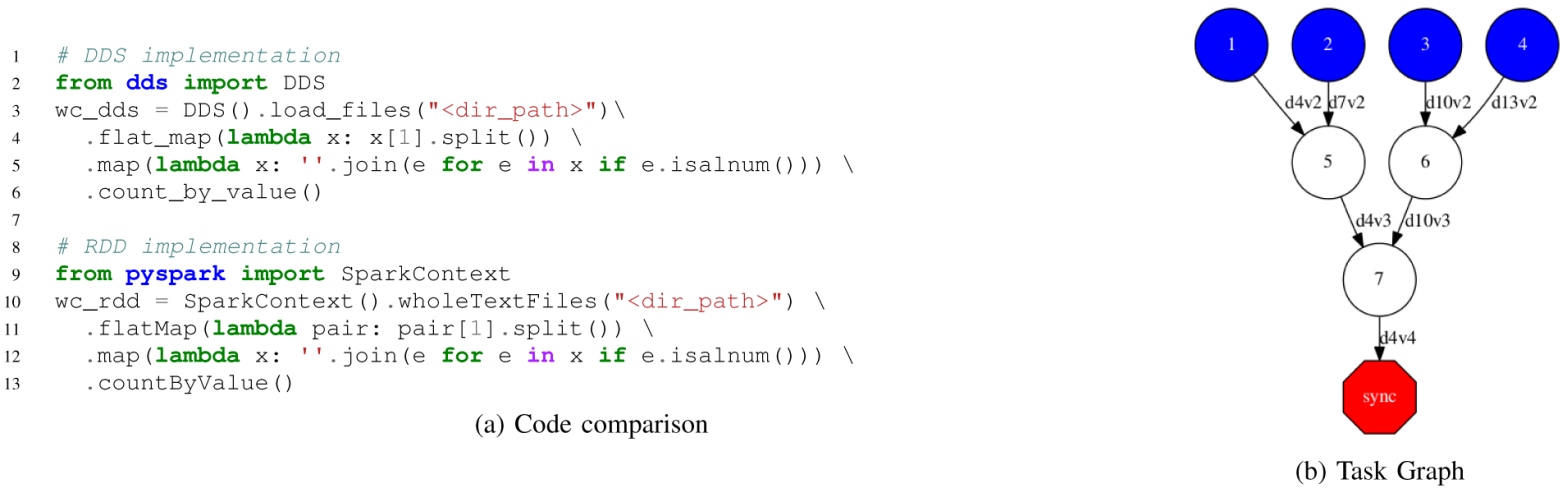
Word Count implemented with DDS.

To overcome the issues mentioned above, we have designed an optimized version of the DDS library. The main goal of the new approach is to minimize the number of generated tasks. First of all, we improved the data loader methods and implemented them in a way that there are no particular tasks only to load data. Partitions are loaded inside transformation or action tasks. Moreover, we also changed the behaviour of transformation tasks. Contrary to the naive implementation, we have incorporated the idea of combining consecutive transformation tasks and running them within a single task. In other words, multiple transformation methods are wrapped together and executed inside the same task that loads the partition. Furthermore, the invocation of that kind of transformation tasks is triggered when one of the action methods is called. Additionally, since multiple tasks are combined into one, the runtime overhead of managing multiple small tasks has been lowered. Figure 2b depicts the optimized version’s behaviour, where we can observe the differences referred to earlier. Looking at the examples illustrated by Figure 2, in the optimized solution there is only one task per partition added to the DAG for the first three operations – data load and transformations 1 and 2 – whereas, in the naive solution, there would be three. Likewise, transformations 3 and 4 are combined and executed within a single task before the final synchronization. Finally, Figure 3 shows the code and the graph of a Word-Count application. In the code part, we compare the DDS and Spark implementations, where we can see the changes are minimal. In the generated graph, we can see the load and maps are executed in the first tasks (blue circles), and the *count by value* is implemented as a reduction in two phases (white circles).

Following pseudo-algorithm steps can be helpful to summarize the internal logic of the DDS:

1. initialize DDS
2. distribute the INPUT
3. while more transformation functions, wrap the functions
4. run combined transformation function(s) on each partition
5. run action function(s)
6. repeat 3, 4, 5 if necessary
7. collect the results.

The most critical part to notice is that in the third step multiple transformation functions are combined and executed together on each partition. DDS handles it with the help of its Child object which is hidden from the user. Every time when the user calls a transformation function, the function is wrapped and passed to a Child DDS object. At the invocation of an action function, Child DDS object goes through the list of the transformation functions from its parents and applies them on each partition.

## IV. Implementation

To validate the proposed methodology we have implemented a prototype of the DDS on top of the PyCOMPSs programming model. Next paragraphs provide an overview of PyCOMPSs and the details of the DDS prototype implementation.

PyCOMPSs ^19^ is the Python binding of the COMPSs framework ^12^ that facilitates the development of parallel computational workflows for distributed infrastructures. It offers a programming model based on sequential development – the application is a plain sequential Python script – where the user annotates the functions to be run as asynchronous parallel tasks. This decorator also contains a description of the function parameters, such as type and direction, which are vital for building the dependency graph. In this graph, tasks are represented as nodes and data dependencies between tasks as edges. At execution time, asynchronous tasks are created for each decorated function and forwarded to the COMPSs Runtime which handles data dependency analysis, task scheduling and data transfers. The task creation is performed in an asynchronous way, and once the runtime has added a given task to the dependency graph, the execution of the main Python code continues, possibly generating new tasks. With this aim, PyCOMPSs manages future objects: a representant object is immediately returned to the main program when a task is invoked. A future object returned by a task can be involved in subsequent asynchronous task calls and PyCOMPSs will automatically find the corresponding data dependencies without requiring to wait for the actual result of the task. PyCOMPSs applications are deployed as master-worker applications, where the master executes the main code and invokes the runtime, and the workers execute the tasks.

**Figure 4.**
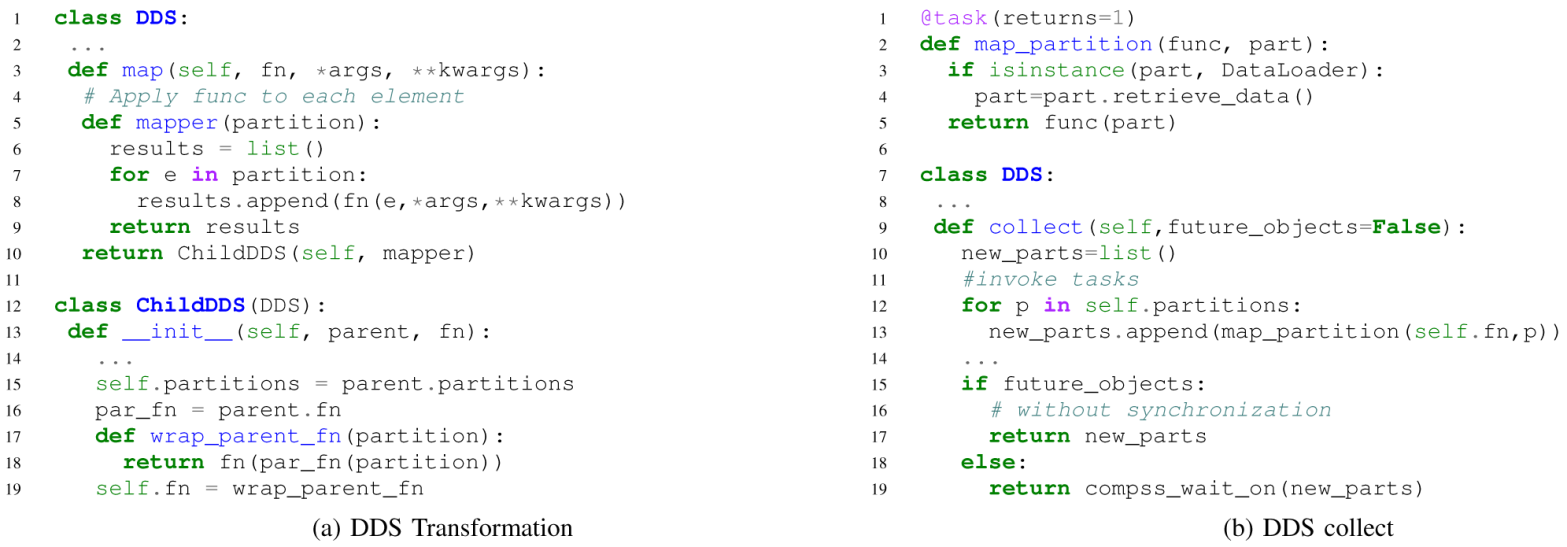
Code snippets of the DDS implementation in PyCOMPSs.

DDS has been implemented on top of PyCOMPSs following the ideas presented in Section III. DA applications normally start with loading some data, then applying several transformation and action functions. Data loader and transformation operations are lazy operations in DDS. When the user calls a data loader function, DDS simply creates one DataLoader object per partition without retrieving the actual data. These DataLoader objects are later sent to the tasks to load the data at execution time. Then, when the user code ’maps’ a transformation to the elements of the DDS, the DDS class creates a helper ’mapper’ function as shown in Figure 4a. The helper ’mapper’ is meant to take the user’s transformation function as a parameter, and apply it to each element inside the partition. Thus, it is equivalent to the user’s transformation operation with the only difference that the ’parameter’ is the partition itself, instead of the elements. Moreover, every new transformation method creates a new ChildDDS object. A ChildDDS object is a DDS object that inherits its parent’s transformation function and wraps it with the new-coming transformation. Following this structure, DDS can combine several transformations and operate them at once. The invocation of the combined transformations happens when a ’collect’ or any other action method is called. As detailed in Figure 4b, DDS creates one ’map partition’ task per partition, provided with a DataLoader object and wrapped transformations. At execution time, each ’map partition’ task loads its own part of the initial data by calling DataLoader’s ’retrieve data’ method and then performs the combined transformations. Through the ’future objects’ parameter of the ’collect’ method, results of those tasks can be synchronized in the main program, or directly passed to other DDS or PyCOMPSs tasks.

## V. Evaluation

We have evaluated our approach to demostrate that the prototype is valid for implementing integrated HPDA applications and does not underperform current Big Data framework such as PySpark. The results presented in this section have been obtained using the MareNostrum 4 (MN4) Supercomputer where each node has two Intel®Xeon Platinum 8160 (24 cores at 2.1 GHz each) and 96 GB of main memory ^1^. Regarding the software, we have used COMPSs version 2.8 ^2^ for the DDS executions, and for PySpark executions, we have used Spark version 3.0.0 ^3^ in standalone mode on MareNostrum 4 with a basic configuration to fully exploit the cluster nodes. In all cases total number of threads per node were 48 and results are average of 3 repetitions per experiment.

### A. HPDA Integrated application

**Figure 5.**
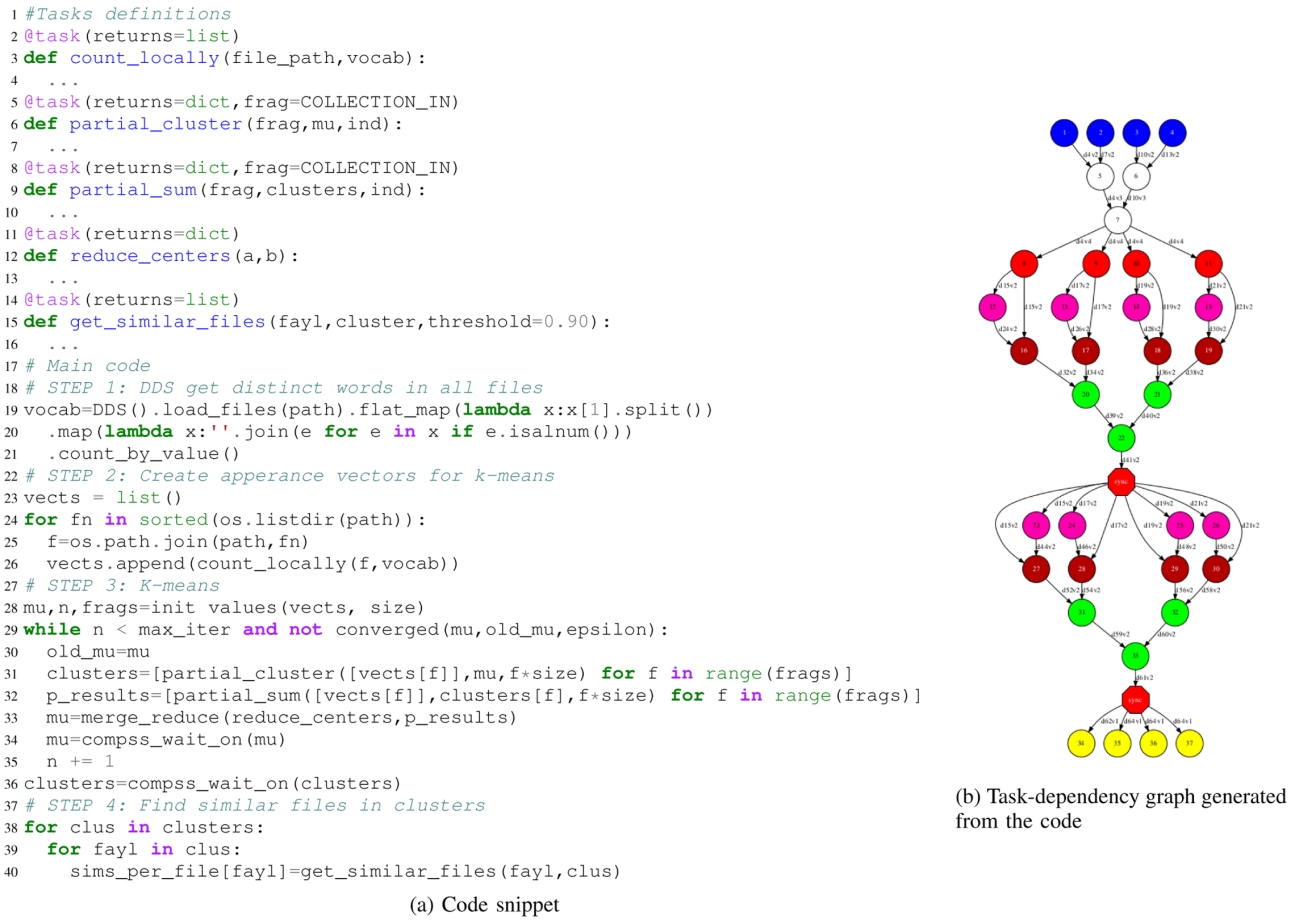
HPDA integrated application. Blue and white circles represent DDS tasks, red circles are count locally tasks, pink, red and green circles are the k-means algorithm tasks, and yellow circles correspond to get similar files tasks.

**Figure 6.**
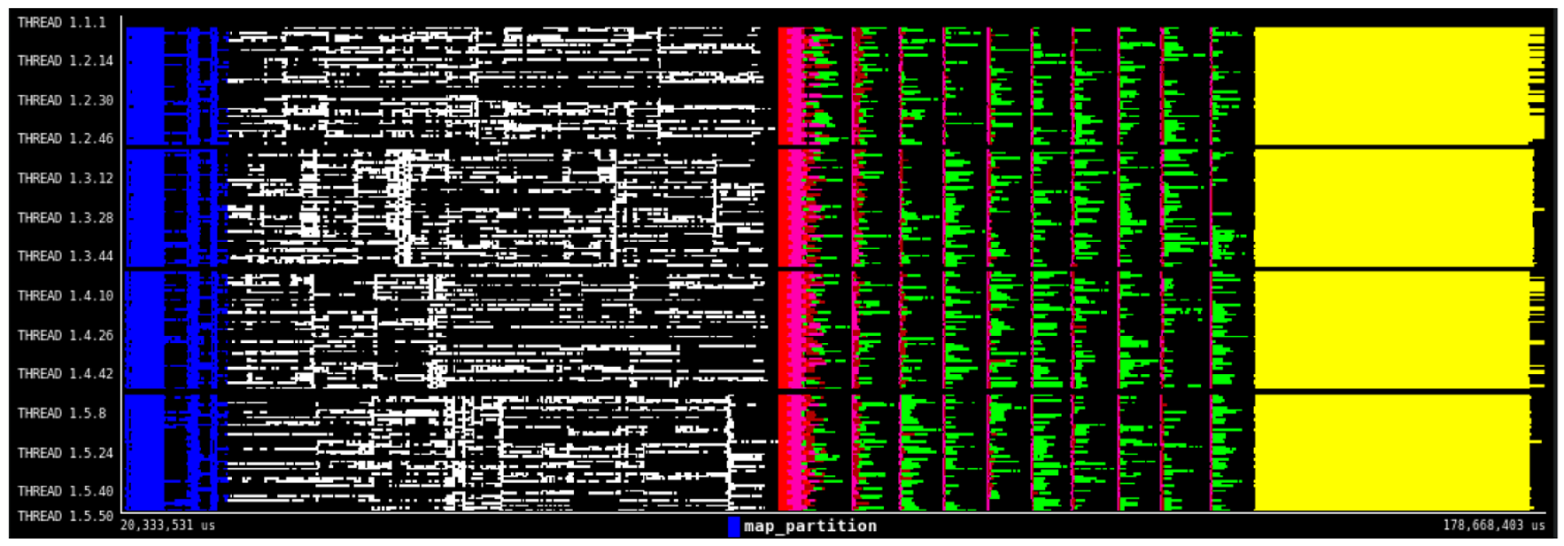
HPDA application execution trace. Y-axis shows the computing nodes threads and x-axis show the execution time. Each color line represents a task executed in a thread.

To demonstrate that our approach can seamlessly integrate HPDA, we have implemented an application to detect similar documents. This application consists of different phases which combine multiple algorithms. Figure 5a shows a code snippet and the task dependency graph generated for the mentioned application ( Figure 5b). It starts with a DA part generating a list containing all the words from the input files (vocabulary on 20). It can be easily retrieved using DDS methods such as ’load-files-from-dir’, ’map-partition’, and ’distinct’. For the second step, we pass this vocabulary to regular PyCOMPSs tasks to generate an appearance matrix for each file in the initial dataset. In these matrices, columns represent the words in alphabetical order, and values are their occurrences. Later on, in the third step, we run a distributed K-Means algorithm with the appearance matrices as input to detect clusters of files with a closer keyword affinity. Once we have the clusters, new tasks are called to compare each file with its cluster ’neighbours’, and identify the most similar files. File comparisons are performed with the ’spaCy’ Python module ^4^. Finally, Figure 6 shows the execution trace automatically generated by PyCOMPSs. In this view, we can see how the PyCOMPSs runtime is scheduling and managing the execution of the DDS-generated tasks together with the rest of the application tasks. Following the same color coding as the DAG from Figure 5, the blue and the white lines represent tasks generated by the DDS. The next lines in the trace are of the "count_locally" function which has a "@task" decorator from PyCOMPSs. Different executions of those tasks are done in the worker node respecting the data locality from previous DDS tasks, thus avaoiding extra inter-node transfers. After that, pink, brown and green lines appear representing execution of the "K-Means" which is the last step of the HPDA application. This example demonstrates how DDS can seamlessly integrate DA algorithms together with other types of computations in the same application.

### B. Performance Comparison Spark’s RDD versus DDS

**Figure 7.**
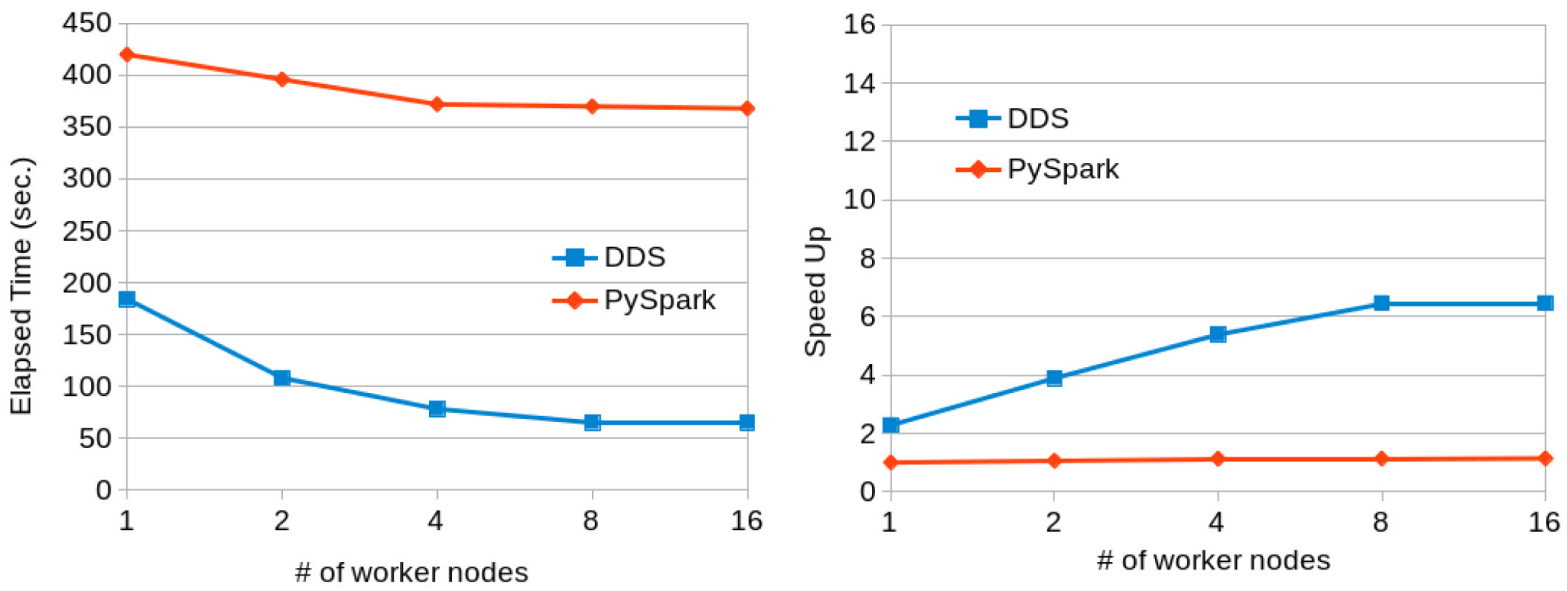
Word Count executions comparison for Gutenberg dataset (~ 80 GB).

**Figure 8.**
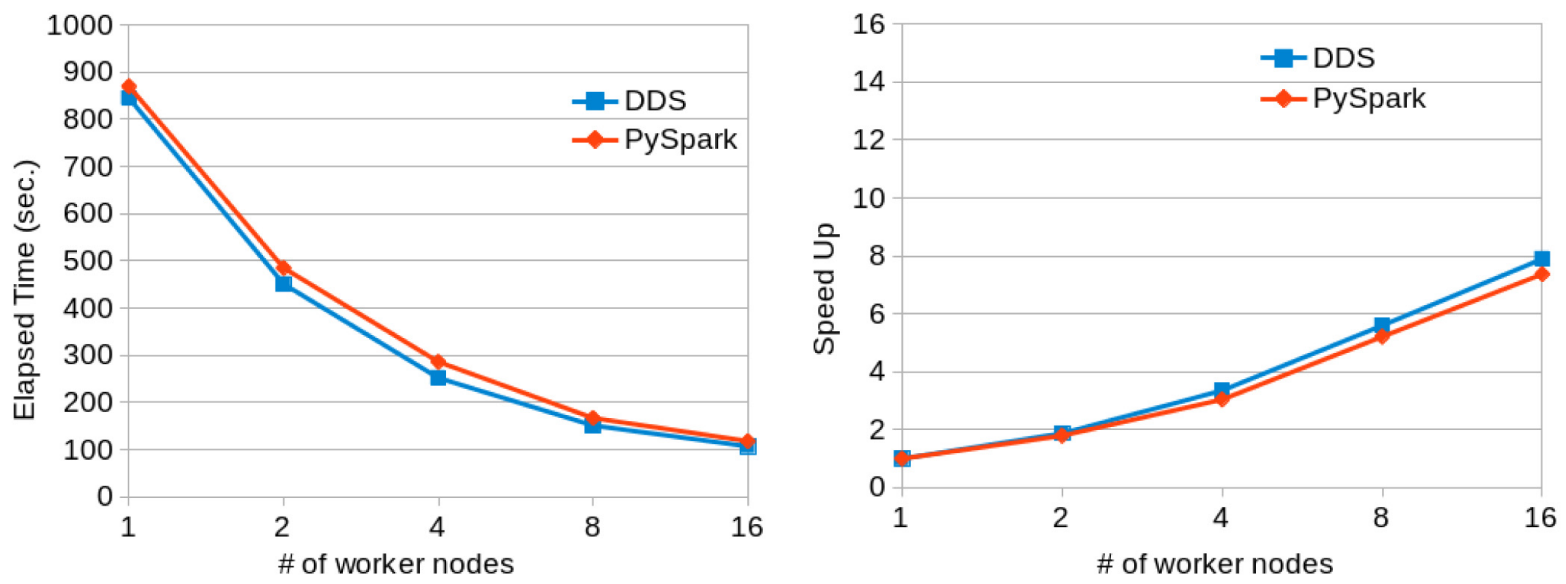
Word Count executions with Lorem Ipsum dataset (100GB).

We have compared the performance of DDS with PySpark’s RDD with different benchmark applications: the Word Count, Terasort and Transitive Closure. The first one is the Word Count program that consists of two phases; in the first step, files are read from the disk as partitions and words of each file are counted locally. After that, local results are being merged within multiple reduce tasks. For these experiments, we run the Word Count with the classic books in English included in the Gutenberg Project ^5^ as input. Figure 7 shows the execution times for PySpark and DDS using a variable number of nodes. We can see that DDS performs better than PySpark but both of them are not scaling well due to the characteristics of the dataset (the speedup is computed using as baseline the execution time with PySpark and one worker node). The reduce part takes most of the time and it does not scale linearly with the number of resources. Figure 8 shows the same execution with the Lorem-Ipsum dataset. This dataset uses a reduced number of words and a better scalability is achieved in both cases, with slightly better results for DDS.

**Figure 9.**
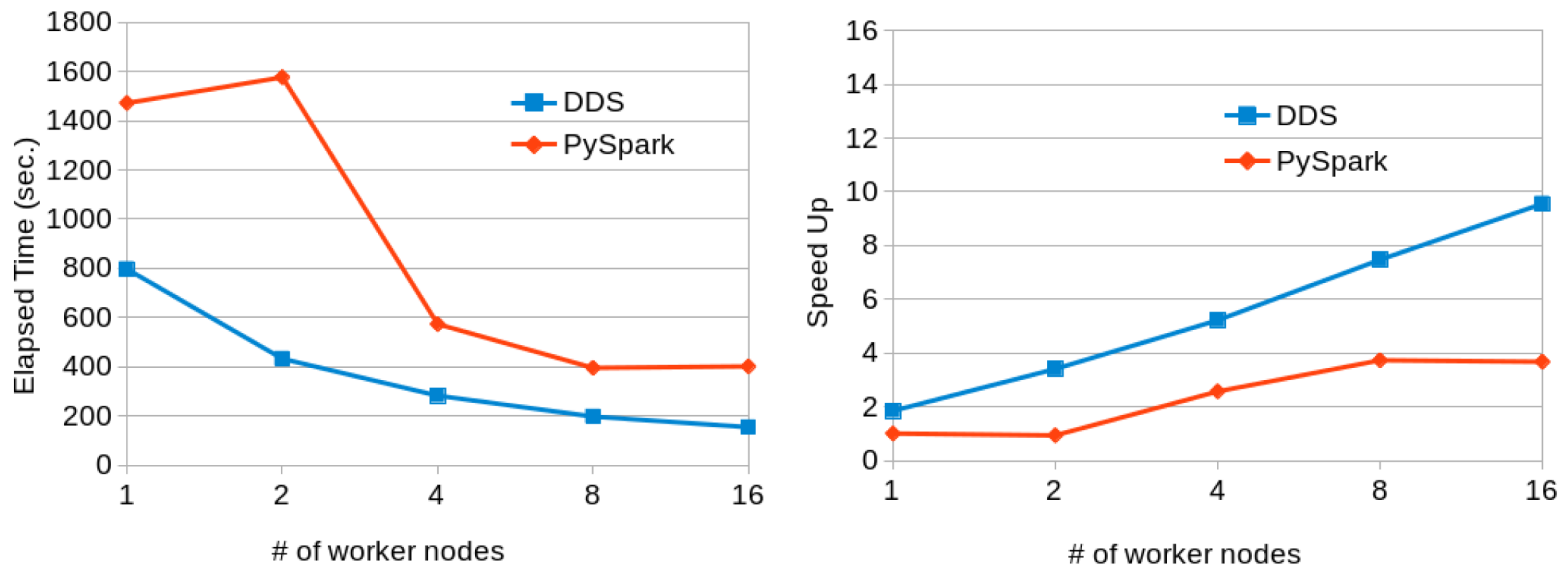
TeraSort executions comparison (200GB dataset).

The second benchmark we used for performance comparison is TeraSort. Even though the original algorithm widely used for benchmarks consists of two additional steps for data generation and validation, in our experiments, we only ran the sorting phase. TeraSort is tested with datasets containing key-value pairs where keys are 10 bytes of data to be sorted, and values are 90 bytes of data corresponding to each key. The algorithm’s idea is to create multiple buckets for different key ranges with non-overlapping bounds and use a ‘divide and conquer’ strategy for the sorting process. First, each of the original data partitions assigns its elements to the corresponding buckets. When all the data has been distributed in the buckets, buckets are locally sorted. Considering that sorting the buckets by their bounds will also sort the whole dataset, no further computation is required after that step. Figure 9 shows the execution times and scalability results obtained when running the Terasort application with PySpark and DDS in a variable number of MN4 nodes. Again, DDS has better performance than PySpark and also better scalability.

**Figure 10.**
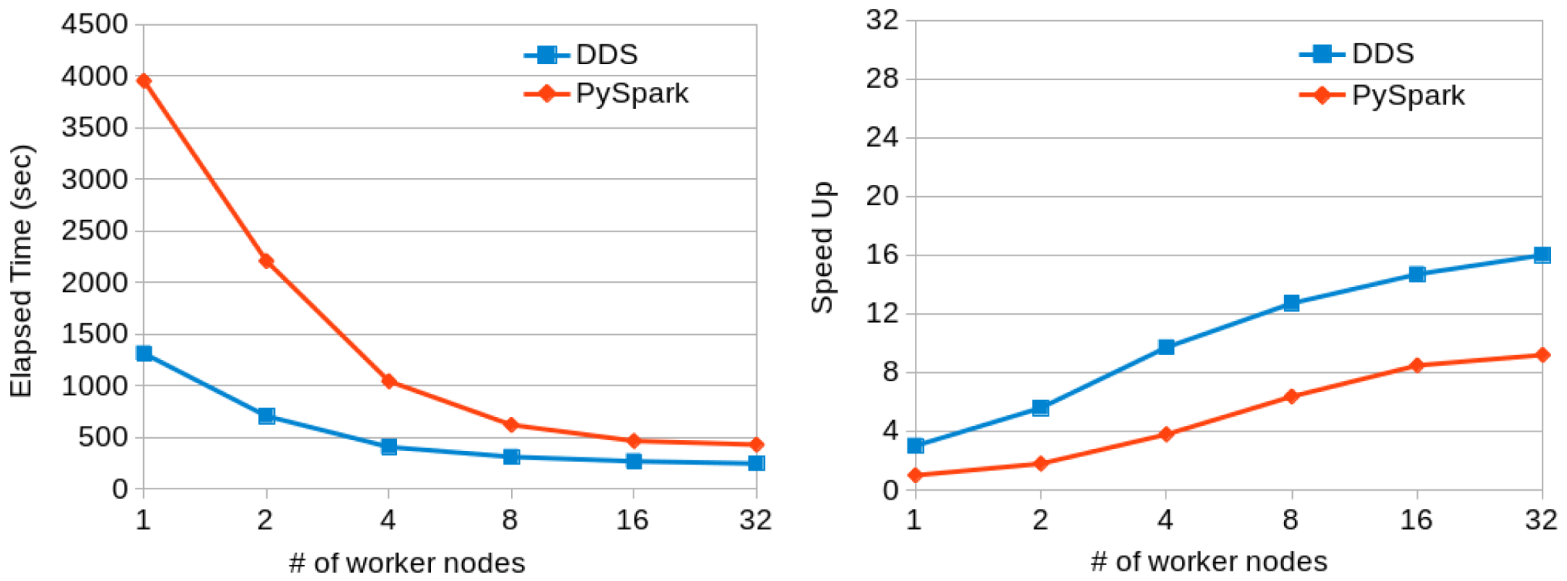
Transitive Closure executions comparison (15GB dataset).

As the last example, we have implemented the Transitive Closure (TC) which is a simple reachability matrix within a given graph. The input data are ”source” and ”destination” nodes for each vertex, and the algorithm builds a final matrix where all possible connections are represented. In our implementation, we followed the PySpark’s approach where in each iteration paths grow by one edge. For example, for edges (x,y) and (y,z), after the first round, (x,z) edge will be added to the discovered paths. The loop stops when the number of discovered edges does not change at the end of the iteration. Figure 10 shows the results obtained when running the program with 15-GB dataset with PySpark and DDS in a variable number of MN4 nodes. In this case, while DDS has better performance than PySpark, PySpark has slightly better scalability. However, not sufficient to perform better than DDS. The time achieved by Pyspark with 16 nodes can be achieved by DDS with just 4 nodes.

## VI. Conclusion

This paper has presented a methodology to develop integrated HPDA applications where data analytics (DA) and HPC algorithms are combined on top of the task-based programming model. To achieve seamless integration of these two types of codes, we have presented the distributed data set (DDS) library, which implements the main used DA transformations and actions on top of a task-based programming model. From the different DA transformation and actions, the DDS produces a task graph whose input results can be seamlessly integrated with the rest of the task-based parallel codes, creating a composed task-dependency graph which is managed by the task-based runtime as a single application. A prototype of the DDS has been implemented on top of the PyCOMPSs programming model and its validation has been focused on two aspects. On one hand, we have developed an HPDA application where a DA algorithm is combined with a K-means clustering algorithm and an algorithm to find text similarities. On the other hand, we have evaluated the performance of our DDS implementation comparing it with PySpark. Results from the evaluation demonstrate that DDS performs better than PySpark with similar scalability and DA codes can be seamlessly integrated with other task-based parallel codes allowing the users to create complex HPDA applications. Finally, although configuration constraints and execution on containers are supported in PyCOMPSs, current version of DDS does not provide these options. Nevertheless, they can be implemented in the future versions in order to fully benefit from PyCOMPSs' features.

DDS performs better than PySpark with similar scalability and DA codes can be seamlessly integrated with other task-based parallel codes allowing the users to create complex HPDA applications.

## Data Availability

Classical books from Gutenberg project that were used for Word Count experiments ^21^. News articles that were used for HPDA application can be accessed on 20. Other datasets can be generated using generators on 22– 24.

## VIII. Software availlability

Archived source code at time of publication on 25 Licence: Open Access
