# Supplementary material for: DDS: integrating data analytics transformations in task-based workflows
Source: Open Res Eur. 2023 Apr 11;2:66. Originally published 2022 May 25. [Version 2] doi: 10.12688/openreseurope.14569.2 (PMC10446079; doi:10.12688/openreseurope.14569.2)
Supplement: Peer review history

# Peer review: Reviewer response for version 2

**DOI:** 10.21956/openreseurope.17120.r31123

**Published:** 2023-05-18

**Authors:** Peggy Lindner

**Affiliations:** 1 Department of Information & Logistics Technology, University of Houston, Houston, TX, USA

The authors have addressed the issues that I listed in my first review.

Is the study design appropriate and does the work have academic merit?

Yes

Is the work clearly and accurately presented and does it cite the current literature?

Yes

If applicable, is the statistical analysis and its interpretation appropriate?

Not applicable

Are all the source data underlying the results available to ensure full reproducibility?

Yes

Are the conclusions drawn adequately supported by the results?

Yes

Are sufficient details of methods and analysis provided to allow replication by others?

Partly

Reviewer Expertise:

Workflows for Data Science applications

I confirm that I have read this submission and believe that I have an appropriate level of expertise to confirm that it is of an acceptable scientific standard.

# Peer review: Reviewer response for version 2

**DOI:** 10.21956/openreseurope.17120.r31122

**Published:** 2023-04-17

**Authors:** Rafael Ferreira da Silva

**Affiliations:** 1 Oak Ridge National Laboratory, Oak Ridge, TN, USA

The authors have successfully addressed all of the minor concerns I had, and as a result, the paper is now ready for indexing.

Is the study design appropriate and does the work have academic merit?

Yes

Is the work clearly and accurately presented and does it cite the current literature?

Partly

If applicable, is the statistical analysis and its interpretation appropriate?

Yes

Are all the source data underlying the results available to ensure full reproducibility?

Yes

Are the conclusions drawn adequately supported by the results?

Yes

Are sufficient details of methods and analysis provided to allow replication by others?

Partly

Reviewer Expertise:

Distributed Computing, HPC, Scientific Workflows

I confirm that I have read this submission and believe that I have an appropriate level of expertise to confirm that it is of an acceptable scientific standard.

# Peer review: Reviewer response for version 1

**DOI:** 10.21956/openreseurope.15731.r29377

**Published:** 2022-07-12

**Authors:** Rafael Ferreira da Silva

**Affiliations:** 1 Oak Ridge National Laboratory, Oak Ridge, TN, USA

This paper provides a methodology for enabling the development of high-performance data analytics (HPDA) applications using task-based programming models (TBPMs). Specifically, authors propose a distributed data set (DDS) approach for implementing efficient data transformations and actions using task-based parallelism. The proposed methodology is evaluated using benchmark applications (word count, TeraSort, and transitive closure), and compared to the current widely used PySpark approach. Overall, DDS yields relatively significant better performance when handling data analytics applications.

 General comments:

- The proposed methodology could be (to some extent) compared to in situ processing technologies. I would recommend authors to provide a brief discussion (potentially on the introduction and related work sections) about in situ data analytics and how it differs from the proposed methodology.
- Could authors provide a description of the proposed method using pseudo-algorithms?
- The experimental evaluation would benefit from some additional experiments/discussions to demonstrate how the DDS yields better resource management. Maybe authors could leverage some efficiency metrics?
- Experimental results are based on strong scaling, which demonstrates relevant improvement using DDS when compared to PySpark. The paper could also benefit from a weak scaling experiment in which results may lead to conclusions that could clearly provide insights about PySpark’s low performance.

Minor comments:

- Consider replacing “What is more” by something like “Furthermore” or “Additionally”.
- Typo in first paragraph section 3: “such as the defined” ==> “such as the ones defined”.
- In Figs 7-10, maybe you can only show speed up, as the elapsed time plots provide relatively similar information.
- I would suggest authors to replace the term “master-worker” by “coordinator-worker” in an attempt to suppress oppressive language (see more at https://tools.ietf.org/id/draft-knodel-terminology-00.html#rfc.section.1.1).

Is the study design appropriate and does the work have academic merit?

Yes

Is the work clearly and accurately presented and does it cite the current literature?

Partly

If applicable, is the statistical analysis and its interpretation appropriate?

Yes

Are all the source data underlying the results available to ensure full reproducibility?

Yes

Are the conclusions drawn adequately supported by the results?

Yes

Are sufficient details of methods and analysis provided to allow replication by others?

Partly

Reviewer Expertise:

Distributed Computing, HPC, Scientific Workflows

I confirm that I have read this submission and believe that I have an appropriate level of expertise to confirm that it is of an acceptable scientific standard, however I have significant reservations, as outlined above.

# Response

**Published:** 2023-03-31

**Authors:** nihad mammadli

**Affiliations:** Barcelona Supercomputing Center, Spain

First of all, thank you very much for your helpful comments, we have updated the document based on majority of your comments. However, even though we are aware that some more experiments would be useful to enhance the Evaluation section, unfortunately the project is already over and we do not have resources to run new experiments. We also believe that the evaluations that are already provided are sufficient to conclude the ideas.

# Peer review: Reviewer response for version 1

**DOI:** 10.21956/openreseurope.15731.r29376

**Published:** 2022-07-07

**Authors:** Florina M. Ciorba, Ahmed Eleliemy

**Affiliations:** 1 Department of Mathematics and Computer Science, University of Basel, Basel, Switzerland; 2 University of Basel, Basel, Switzerland

Summary

 HPC Workflows use specific parallel programming models, while data analytic (DA) applications mainly exploit data transformations. Among those HPC parallel programing models, task-based programming models (TBPMs) are very efficient for HPC workflows. The authors proposed a methodology to develop HPDA applications on top of TBPMs. The authors proposed a library called Distributed Data Set (DSS) that implements data transformations and actions (such as the defined in RDD’s Spark’s API) using the PyCOMPs task-based library. The authors implemented multiple benchmarks, and the results showed that the proposed library outperforms the corresponding version implemented via PySpark.

Strengths

 The authors close a gap in the state of practice for developing HPDA applications.

 The proposed library (DDS) offers similar APIs to well-known data processing frameworks (Spark), i.e., applications will require minimal changes.

 The manuscript is well written and structured.

Weaknesses

 In the Evaluation section, the authors report the obtained results rather than discussing and giving insights on why these results, i.e., the Evaluation Section leaves the reader with more questions than insights about the work.

More details and suggestions for improvements

 A detailed section about how the library internally works would be appreciated. What tasks are fusible and why?

 Evaluation section: how many threads are configured per worker node? Do Spark and COMPs have the same number of threads per node?

 More experiments would be beneficial that take into consideration the option of oversubscription for the number of threads per node (in the case of Spark).

 The authors attributed the performance superiority of their library to the ability to combine multiple tasks and have one data loader task. Please time individual parts within the code for both implementations, DDS and PySpark, to confirm your performance superiority claims.

 Are the reported results based on one or several repetitions per experiment? Reporting this will increase the trustworthiness statistically.

 Figures 7 and 8 show the performance of DSS and PySpark implementations of the Word Count benchmark. The obtained results require clarifications, i.e., both implementations have the same performance in the case of the Ipsum dataset, while DSS extremely outperforms PySpark in the case of Gutenberg.

 Irrespective of the experiment, the performance of PySpark implementations is poor for a few nodes (1, 2, 4). But, it seems that PySpark implementations scale better than DSS. The performance of PySpark implementations improves beyond 4 nodes. Would it be better than DSS if we go beyond 32 nodes?

 Why do specific experiments use 32 nodes while others use up to 16 nodes?

 It is important to also include the limitations of the proposed DSS, at least those inherited from COMPs.

 How does the DDS approach differ from recent approaches such as DAPHNE (https://www.cidrdb.org/cidr2022/papers/p4-damme.pdf)? ^1^

Is the study design appropriate and does the work have academic merit?

Yes

Is the work clearly and accurately presented and does it cite the current literature?

Partly

If applicable, is the statistical analysis and its interpretation appropriate?

Partly

Are all the source data underlying the results available to ensure full reproducibility?

Yes

Are the conclusions drawn adequately supported by the results?

Yes

Are sufficient details of methods and analysis provided to allow replication by others?

Partly

Reviewer Expertise:

High performance computing, high performance data analysis, scheduling and load balancing, tasking

We confirm that we have read this submission and believe that we have an appropriate level of expertise to confirm that it is of an acceptable scientific standard.

## References

[1] : DAPHNE: An Open and Extensible System Infrastructurefor Integrated Data Analysis Pipelines. *Conference on Innovative Data Systems Research* .2022; Reference source

# Response

**Published:** 2023-04-05

**Authors:** nihad mammadli

**Affiliations:** Barcelona Supercomputing Center, Spain

For all experiments we have measured the execution times of Python code. It is true that timing individual parts would strengen our claims, it would be possible for us only to include different timers in our API to achieve this goal, we wouldn't be able to do the same with Spark's RDD. Thus, we only measured the execution times which can be done with both frameworks.

# Peer review: Reviewer response for version 1

**DOI:** 10.21956/openreseurope.15731.r29379

**Published:** 2022-06-27

**Authors:** Peggy Lindner

**Affiliations:** 1 Department of Information & Logistics Technology, University of Houston, Houston, TX, USA

The paper presents an approach to combining data analytical tasks with high-performance computing. The introduction provides a good, generalized background of the topic that quickly gives the reader an appreciation of the applicability of integrating a task-based programming model suitable for HPC environments with data analytics algorithms. The authors present the motivation and background for such an approach very well. The related work section is clear and sufficient.

 The methodology section explains in an effective way how the distributed data set approach can be optimized for generated tasks. The implementation details are laid out detailed enough to follow the approach presented.

 The evaluation section demonstrates a prototypical application implementation which is also used for performance evaluation.  It needs minor revisions and clarifications:

1. I would not call Part A) an “experiment” (see the first sentence in Part V. “ *We have performed two experiments …”*.) Please clarify the language used to introduce the evaluation section.
2. Please add some better explanations for the reader to describe the trace file (figure 6). While I appreciate the inclusion of the trace file, it would be good to refer to the color coding to tell readers which parts refer to “ *see how the PyCOMPSs runtime is scheduling and managing the execution of the DDS-generated tasks together with the rest of the application tasks*.”
3. Please add more details to the summary of Part A “ *This example demonstrates how DDS can seamlessly integrate DA algorithms together with other types of computations in the same application.* “
4. The performance evaluation section should be enhanced with further details about the sample sizes used in the experiments (e.g., number of books/words etc.). Currently, it’s hard to judge the experiments for their applicability to real-world scenarios (the selected algorithms are a good representation).

The conclusion section summarizes the presented work very well but is missing a discussion about limitations and/or future work.

 Data Availability section: “News articles that were used for HPDA application can be accessed on ^20^. “ It is not very clear that this dataset is used in the prototype implementation example. Please mentioned the usage in the HDPA integrated application section.

 I was not able to access the source code used for the evaluation. The source code link given under [12] points to

https://github.com/ElsevierSoftwareX/SOFTX-D-15-00010 which is not accessible. That has to be fixed. The link for COMPSS 2.8.1 is fine.

 Minor formatting issues: the first paragraph in the conclusion breaks up in the middle of a sentence. “ *Results from the evaluation demonstrate that*

*DDS performs better than PySpark with similar scalability* …”

Is the study design appropriate and does the work have academic merit?

Yes

Is the work clearly and accurately presented and does it cite the current literature?

Yes

If applicable, is the statistical analysis and its interpretation appropriate?

Not applicable

Are all the source data underlying the results available to ensure full reproducibility?

Yes

Are the conclusions drawn adequately supported by the results?

Yes

Are sufficient details of methods and analysis provided to allow replication by others?

Partly

Reviewer Expertise:

Workflows for Data Science applications

I confirm that I have read this submission and believe that I have an appropriate level of expertise to confirm that it is of an acceptable scientific standard, however I have significant reservations, as outlined above.
